# Nurturing the reading brain: home literacy practices are associated with children’s neural response to printed words through vocabulary skills

**DOI:** 10.1038/s41539-021-00112-9

**Published:** 2021-12-03

**Authors:** Cléa Girard, Thomas Bastelica, Jessica Léone, Justine Epinat-Duclos, Léa Longo, Jérôme Prado

**Affiliations:** Lyon Neuroscience Research Center (CRNL), INSERM U1028 - CNRS UMR5292, University of Lyon, 69500 Bron, France

**Keywords:** Language, Reading

## Abstract

Previous studies indicate that children are exposed to different literacy experiences at home. Although these disparities have been shown to affect children’s literacy skills, it remains unclear whether and how home literacy practices influence brain activity underlying word-level reading. In the present study, we asked parents of French children from various socioeconomic backgrounds (*n* = 66; 8.46 ± 0.36 years, range 7.52–9.22; 20 girls) to report the frequency of home literacy practices. Neural adaptation to the repetition of printed words was then measured using functional magnetic resonance imaging (fMRI) in a subset of these children (*n* = 44; 8.49 ± 0.33 years, range 8.02–9.14; 13 girls), thereby assessing how sensitive was the brain to the repeated presentation of these words. We found that more frequent home literacy practices were associated with enhanced word adaptation in the left posterior inferior frontal sulcus (*r* = 0.32). We also found that the frequency of home literacy practices was associated with children’s vocabulary skill (*r* = 0.25), which itself influenced the relation between home literacy practices and neural adaptation to words. Finally, none of these effects were observed in a digit adaptation task, highlighting their specificity to word recognition. These findings are consistent with a model positing that home literacy experiences may improve children’s vocabulary skill, which in turn may influence the neural mechanisms supporting word-level reading.

## Introduction

**Fig. 1:**
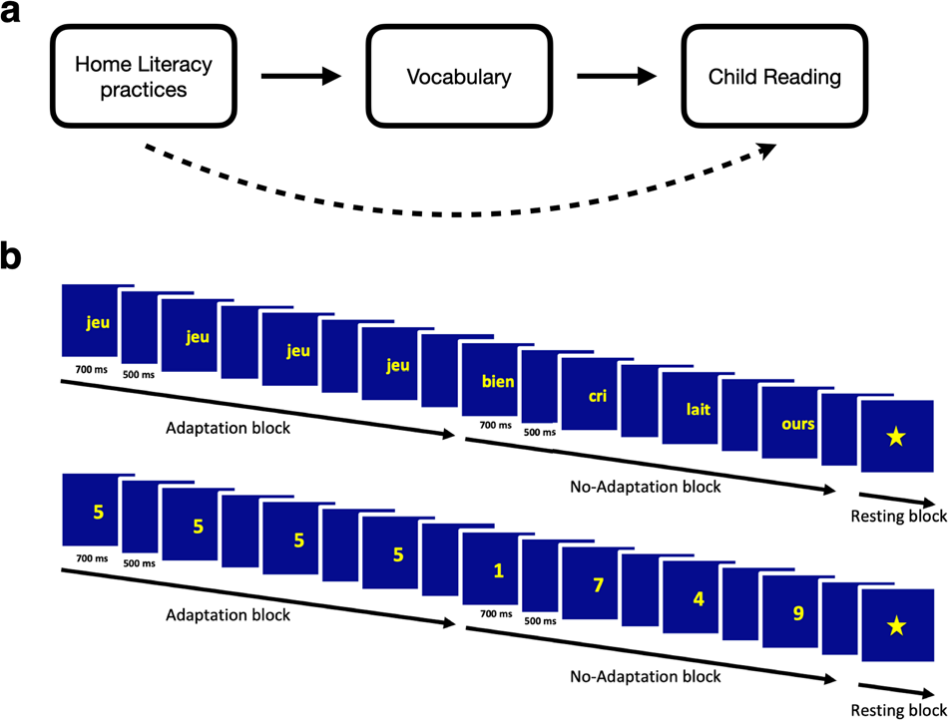
Model and experimental design. **a** Hypothesized model of the relations between home literacy practices, vocabulary and reading. **b** Experimental task. Participants were sequentially presented with words (top) or digits (bottom) that were identical (adaptation blocks) or different (no-adaptation blocks).

Literacy skills are fundamental to children’s personal and professional growth in our society. Yet, children vary greatly in their reading development throughout the school years^1^. Because such individual differences can be observed as early as in the preschool years, it has been argued that the home literacy environment (HLE) may play an important role in scaffolding early literacy skills^2^. The HLE typically refers to the literacy-related interactions, resources, and attitudes that children experience at home^3^. Though the quality of the HLE may be estimated through various measures (e.g., title recognition, reading exposure, language input), it is most often assessed using parental self-administered questionnaires^4,5^. Consistent with a scaffolding role of the HLE for the development of literacy, studies have found that a supportive HLE is associated with both enhanced vocabulary^4,6,7^ and improved reading skills^4,5,8,9^ in children. This has notably led some to hypothesize that the relation between the HLE and children’s reading may be mediated by vocabulary^5,10,11^. In other words, more frequent home literacy activities may improve children’s reading efficiency by fostering vocabulary skills (which are an important foundation for word recognition)^12^ (see Fig. 1a). This also prompted various organizations to call for greater parent and caregiver involvement in home literacy education^13,14^.

What are the neural mechanisms supporting the relation between the HLE and literacy development in children? The first line of evidence comes from studies investigating how differences in children’s brain structure and function may relate to disparities in family socioeconomic status (SES), an index of family status and position in society (often obtained using measures of parental income, education, or occupation). Indeed, differences in SES are well known to be associated with differences in language input and literacy environment^15,16^. Overall, these studies have found that SES is associated with structural and functional differences in brain regions typically associated with literacy. For example, studies have found a positive relation between emergent literacy skills and cortical thickness in left perisylvian regions^17^. However, lower SES has been associated with lesser cortical thickness in these regions^18,19^. Children from disadvantaged families have also less cortical surface area^20^ in left perisylvian regions than more advantaged peers. Finally, these children have been found to have a weaker specialization of the left inferior frontal gyrus (IFG) during a rhyming task^21^. Thus, there is growing evidence that SES is associated with the structure and function of left perisylvian areas in children, a relation that might be mediated by cognitive precursors to reading acquisition, such as vocabulary^22^ (see Fig. 1a).

The second line of evidence comes from recent studies that have more directly investigated the relation between HLE and brain development, either using parental questionnaires or home recordings. For instance, home reading exposure and maternal shared reading quality have been associated with white matter integrity^23^ and left perisylvian activity during story–listening tasks in preschoolers^24,25^. Using home audio recordings, studies have also showed that the quality of conversational experience with parents at home is associated with greater surface area^26^ and structural connectivity^27^ within the left perisylvian system in children, as well as increased activity in the left IFG during a story-listening task^28^. Another study employing parental questionnaires has found that structural connectivity of left perisylvian areas is associated with both HLE and vocabulary development^29^, in line with the idea that both dimensions may interact to account for reading acquisition^22^. Finally, more supportive HLE has also been linked to increased activity in the left IFG during a sound matching task in 5-year-olds^30^. Therefore, in keeping with studies investigating SES, neuroimaging studies that have more explicitly assessed the HLE also suggest that the quality of adult–child literacy interactions at home might affect both structure and function of left perisylvian areas (particularly at the level of the left IFG).

These previous studies, however, only provide indirect evidence that the HLE relates to brain regions associated with word-level reading in children. This is because studies to date have either assessed structural (not functional) properties of the brain of reading children^26,29^ or have only used functional magnetic resonance imaging (fMRI) to measure the brain activity of pre-reading children using auditory tasks that did not involve word recognition^24,25,28,30^. Thus, although one previous study has assessed the relation between SES and brain regions supporting pseudo-word processing^16^, we do not know if the frequency of home literacy practices is associated with fMRI activity during a word recognition task in children. We also do not know whether this relation may be mediated by pre-literacy skills such as vocabulary, as some have hypothesized^22^.

In the present study, we aimed to test whether fMRI activity in areas supporting word recognition may be related to the HLE of elementary school children. To test this, parents of 8-year-olds completed an extensive questionnaire evaluating the frequency of literacy practices shared with children at home. Children’s literacy skills were also assessed using measures of reading fluency and vocabulary. Finally, fMRI activity associated with word recognition was measured using a word adaptation task developed by Perrachione et al.^31^. In this task, participants are passively presented with blocks of visual (written) words that are either identical (adaptation blocks) or different (no-adaptation blocks) (see Fig. 1b). Such fMRI-adaptation paradigms rely on the idea that presenting a given stimulus repeatedly leads to a decrease of fMRI signal in the region that processes this stimulus because firing rates of the neuronal population decrease^32^. Thus, how sensitive a region is to word processing can be measured by comparing no-adaptation to adaptation blocks, i.e., the so-called neural adaptation effect^31^. Critically, this neural adaptation effect appears to be functionally relevant because it is related to reading skills in adults (i.e., it is smaller in dyslexic readers than in typical readers^31^). To compare our findings to that of Perrachione et al.^31^ (who tested English-speaking adults) and therefore assess task replicability^33^, we first identified the network in which a neural adaptation effect was found across the whole brain in our sample of French-speaking children. To increase statistical power^34^ and provide an unbiased estimate of effect sizes, we then employed a hypothesis-driven approach and measured the word adaptation effect in the exact same regions that were found to exhibit a word adaptation effect in Perrachione et al. in typical readers^31^.

We made five predictions. First, we expected that the frequency of home literacy practices would be associated with both vocabulary and reading fluency skills in children, in line with previous behavioral studies^5^. Second, we anticipated replicating and extending Perrachione et al.’s findings by showing word adaptation effects in the regions that were identified in that previous study using the same task in French-speaking children. These were our regions of interest (ROIs). Third, we expected that more frequent home literacy practices would also be associated with greater neural adaptation to words (i.e., a larger difference in fMRI activity between no-adaptation and adaptation blocks) in at least some of the ROIs, reflecting a relation between HLE and fMRI activity associated with word recognition. Fourth, in line with the model shown in Fig. 1a, we hypothesized that the relation between HLE and both reading fluency skills and word adaptation would be mediated by children’s vocabulary skills. Fifth, we expected that none of the above effects would be observed in a control adaptation task in which words were replaced by other types of stimuli. Therefore, we also presented participants with a control adaptation task in which words were replaced by Arabic digits (see Fig. 1b). This allowed us to test whether any of our effects were specific to words (as compared to any kind of symbolic information), thereby also controlling for general perceptual adaptation. Indeed, processing symbolic numbers involves parietal mechanisms that support quantity processing to a much greater extent than the brain circuits of reading^35^.

## Results

### Samples

Demographic information about children and parents can be found in Table 1 and Methods. The behavioral sample consisted of 66 French-speaking children from age 7.52 to 9.22 (mean = 8.46), while the fMRI sample consisted of 44 of these participants (age range = 8.02–9.14; mean = 8.49). SES ranged from relatively low to relatively high in both samples (see Table 1 and Methods for details). Age-normalized scores for reading fluency and vocabulary skills (assessed using the Alouette-R test^36^ and the “vocabulaire” subtest of the NEMI-2 test^37^, see Methods) were in the normal to superior range in both samples. IQ was also in the normal to the superior range. Parental reading scores were in the normal range (see Table 1). The subset of children in the fMRI sample did not differ from children in the behavioral sample in terms of demographics and scores (all *p*’s > 0.27).

**Table 1:** Demographic information and test scores of children and parents.

	Behavioral sample (*n* = 66)				fMRI sample (*n* = 44)			
	Children		Parents		Children		Parents	
Measure	Mean (SD) or *n*	Range	Mean (SD) or *n*	Range	Mean (SD) or *n*	Range	Mean (SD) or *n*	Range
Age (years)^a^	8.46 (0.36)	7.52–9.22	39.53 (4.95)	29–51	8.49 (0.33)	8.02–9.14	39.25 (4.78)	32–51
Sex (female)	*n* = 20	–	*n* = 59	–	*n* = 13	–	*n* = 39	–
Child grade (second grade)^b^	*n* = 20	–	–	–	*n* = 12	–	–	–
Parental education^c^	–	–	3.05 (2.36)	−7–8	–	–	3.41 (2.17)	−1–8
primary	–	–	*n* = 1	–	–	–	*n* = 0	–
secondary	–	–	*n* = 8	–	–	–	*n* = 6	–
undergraduate	–	–	*n* = 33	–	–	–	*n* = 20	–
master or higher	–	–	*n* = 24	–	–	–	*n* = 17	–
Monthly income^d^	–	–	1727 (1078)	500–5500	–	–	1659 (1098)	500–5500
€0–1000	–	–	*n* = 18	–	–	–	*n* = 14	–
€1000–2000	–	–	*n* = 25	–	–	–	*n* = 15	–
€2000–3000	–	–	*n* = 16	–	–	–	*n* = 11	–
€3000–4000	–	–	*n* = 5	–	–	–	*n* = 3	–
€4000–5000	–	–	*n* = 1	–	–	–	*n* = 0	–
€5000–6000	–		*n* = 1	–	–	–	*n* = 1	–
Reading accuracy^e^	105 (8)	86–120	96 (15)	40–115	106 (8)	86–120	95 (16)	40–115
Reading speed^e^	112 (16)	81–155	107 (13)	74–157	114 (17)	81–155	106 (13)	74–147
Vocabulary^f^	9 (2)	4–14	–	–	10 (2)	6–14	–	–
IQ composite^e^	112 (11)	83–135	–	–	113 (12)	83–135	–	–

There was no difference between the behavioral and the fMRI samples in terms of demographics or performance (all *p*’s > 0.27).

^a^All children were 8 years of age, except for one who was younger (7.51) and three who were slightly older (9.06 to 9.22) at the time of testing.

^b^Children were either in second or third grade.

^c^Education level was measured using the number of years pre- or post- high school graduation, with high school graduation set at 0.

^d^As a reference, the median monthly income in France is about €1,700 (Robin, 2019).

^e^Standard score.

^f^Scaled score.

### Behavioral results

**Fig. 2:**
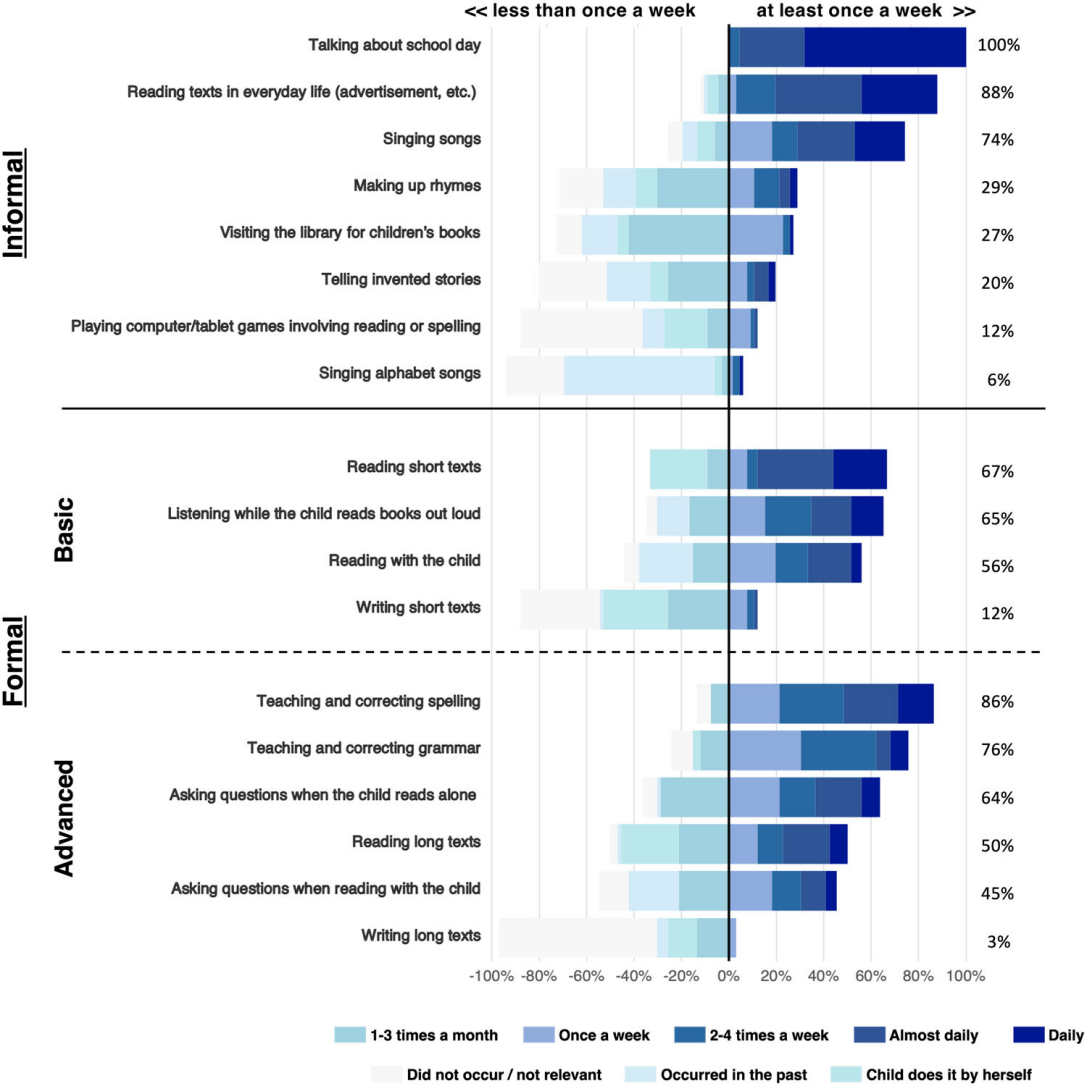
Divergent stacked bar chart showing the frequencies of informal, basic formal, and advanced formal practices among parents (*n* = 66). Percentages on the right indicate the share of parents who engage in each practice at least once a week. Practices are ordered from the most (top) to the least frequent (bottom) for each level of complexity.

Figure 2 shows the frequency of the different home literacy practices reported by parents across items in our questionnaire (parents indicated the frequency of each practice on a six-point rating scale, see Methods). Practices were categorized according to whether they were informal or formal as well as basic or advanced, in line with the previous literature on the HLE^5,38^. As can be seen on Fig. 2, there was large individual variability in the reported frequencies of home literacy practices. Frequencies were then averaged across items within each category to gather separate scores of informal, formal basic, and formal advanced practices. Between parents, average responses on the rating scale ranged from 1 to 3.75 for practices that were considered informal (M = 2.080, SD = 0.548), from 0.5 to 4 for practices that were considered formal but basic for the child’s age (M = 2.140, SD = 0.876), and from 0.670 to 3.840 for practices that were considered formal and relatively advanced (M = 2.006, SD = 0.695). Correlation analyses indicated that neither education nor income was associated with home literacy practices in the behavioral sample (all *p*’s > 0.19, two-tailed). However, education tended to be associated with both basic and formal advanced home literacy practices in the fMRI sample (basic: *r*(64) = −0.259, *p* = 0.090, two-tailed; advanced: *r*(64) = −0.280, *p* = 0.066, two-tailed). As is standard in the literature on the home learning environment, all further analyses were controlled for parental SES (parental income and education).

**Fig. 3:**
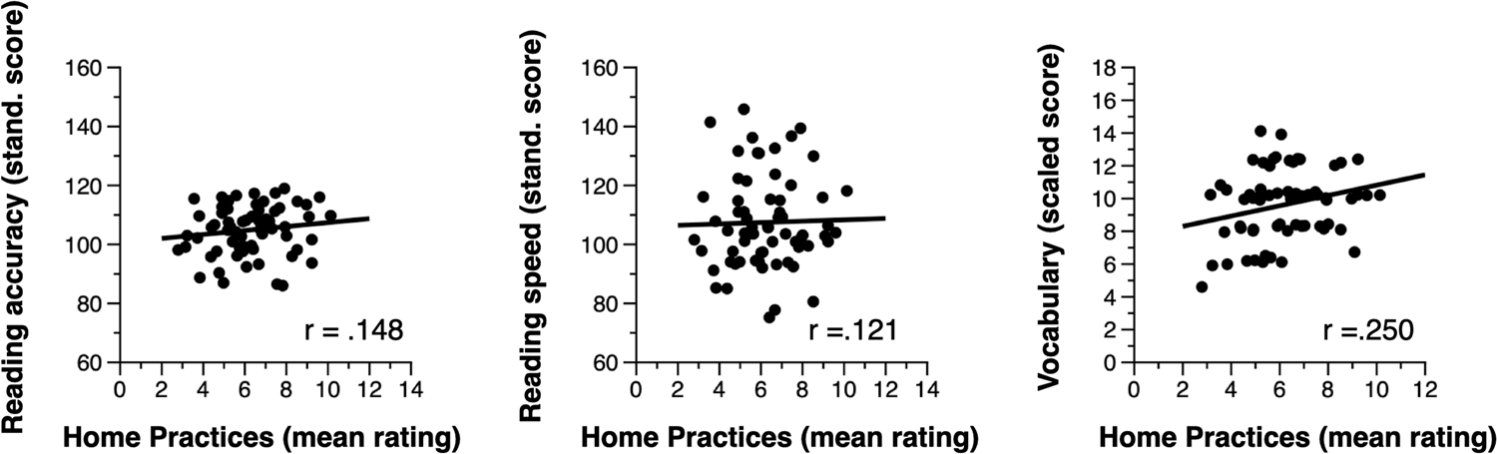
Behavioral results. Scatterplots of child reading accuracy, reading speed, and vocabulary as a function of the overall frequency of home literacy practices (controlled for parental income and education, *n* = 66).

We first investigated the relations between overall home literacy practices (a composite measure calculated by summing the frequency of informal, formal basic, and formal advanced practices) and both reading fluency and vocabulary across this entire behavioral sample. Our first hypothesis was only partly supported. In line with expectations, partial correlations controlling for parental income and education indicated a small positive relation between overall frequency of home literacy practices and child vocabulary (partial *r*(64) = 0.250, *p* = 0.023). However, there was no positive relation between overall frequency of home literacy practices and either child reading accuracy (partial *r*(64) = 0.148, *p* = 0.126) or child reading speed (partial *r*(64) = 0.121, *p* = 0.174) (see Fig. 3). Follow-up analyses revealed that the relation between home literacy practices and vocabulary was relatively consistent across all types and complexity of literacy practices (informal: partial *r*(64) = 0.187, *p* = 0.070, basic formal: partial *r*(64) = 0.182, *p* = 0.075; advanced formal: partial *r*(64) = 0.240, *p* = 0.028). Therefore, neuroimaging analyses focus on the composite measure of the overall frequency of literacy practices.

### fMRI results

We then turned to neuroimaging data and analyzed adaptation effects measured in the word adaptation task (see Fig. 1b) in the fMRI sample. Critically, this task was used in a previous study by Perrachione et al.^31^ (see their Experiment 2B), who investigated neural adaptation to words in a sample of typical (*n* = 23, standardized reading score = 108.0 ± 6.7, see Table 1 in Perrachione et al.^31^) and dyslexic (*n* = 23, standardized reading score = 84.2 ± 6.6, see Table 1 in Perrachione et al.^31^) adult readers. In typical readers, Perrachione et al.^31^ found widespread word adaptation effects in a left-lateralized network, including the temporal (fusiform gyrus, inferior temporal gyrus, superior, and middle temporal gyrus), frontal (inferior frontal gyrus, frontal operculum, premotor cortex, and pre-supplementary motor area), and visual (pericalcarine) cortices (see Fig. 3b and Table S5 in Perrachione et al.^31^). In dyslexic readers, word adaptation effects were weaker and centered around the left inferior frontal cortex (with more limited adaptation in the left middle temporal and inferior temporal cortices).

**Fig. 4:**
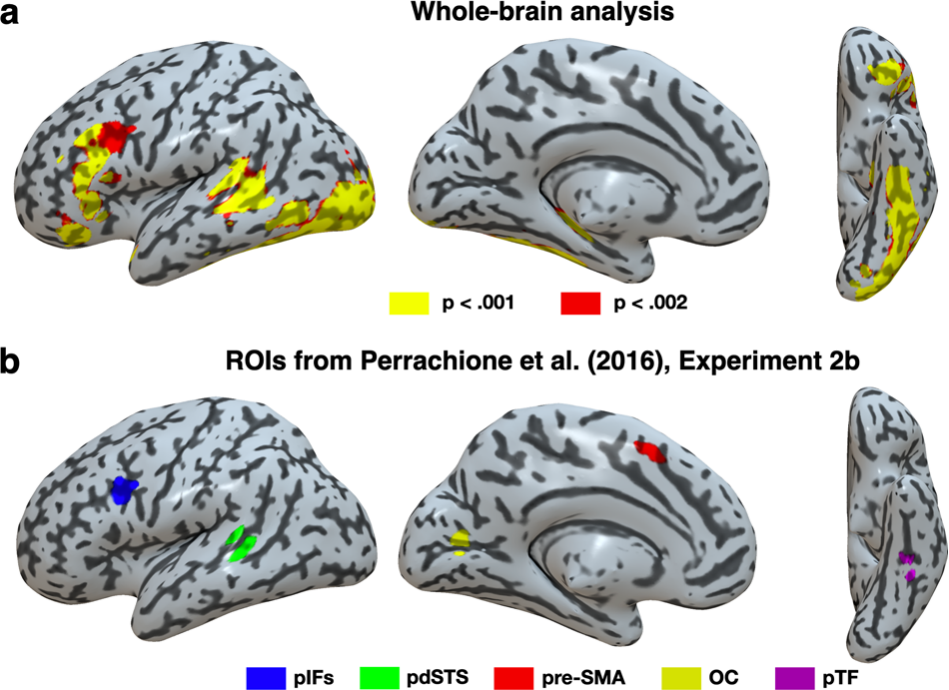
Whole-brain neural adaptation to words and location of ROIs. **a** Brain regions more activated for no-adaptation than adaptation blocks in the word adaptation task across the whole brain. **b** Location of ROIs on lateral and ventral views of an inflated rendering of the cortical surface and an axial slice of an MNI normalized brain. pIFs left inferior frontal sulcus, pdSTS left posterior dorsal superior temporal sulcus, pre-SMA left pre-supplementary motor area, OC left occipital cortex, pTF left posterior temporal fusiform gyrus.

In our sample of French-speaking 8-year-olds, whole-brain analyses (see Methods) showed enhanced word adaptation effects across all participants in a left-lateralized network that was largely similar to that found in typical readers in Perrachione et al.^31^. In our sample, word adaptation effects were also found in the temporal (superior, middle, and inferior temporal gyrus), frontal (notably the left triangular and orbital parts of the inferior frontal gyrus), and visual (inferior occipital gyrus) cortices (see Fig. 4a and Table 2). There was, however, less extensive activation in the left precentral gyrus and left parietal cortex (see Fig. 3b in Perrachione et al.^31^).

**Table 2:** Activation peaks in the whole-brain analysis of the word adaptation task.

Anatomical Location	Cluster size (mm^3^)	MNI coordinates			*t*-score
		X	Y	Z	
Left occipital cortex	7224	−22	−98	−4	6.03
Left inferior temporal gyrus	–	−46	−46	−15	5.35
Left inferior occipital cortex	–	−38	−86	−8	5.31
Left inferior frontal gyrus pars orbitalis	1246	−36	32	−8	4.65
Left inferior frontal gyrus pars orbitalis	–	−42	28	−15	4.18
Left inferior frontal gyrus pars orbitalis	–	−26	30	−12	3.44
Left inferior frontal gyrus pars triangularis	840	−52	26	16	4.61
Left superior temporal gyrus	938	−58	−40	6	4.50
Left middle temporal gyrus	–	−62	−48	6	4.43

*MNI* Montreal neurological institute.

Given the similarity between the brain regions showing a word adaptation effect for typical readers in Perrachione et al.^31^ and in our study (see above), we used the activation peaks for typical readers reported in Table S5 of Perrachione et al.^31^ to define regions of interest (ROIs) in the present study (see Methods). Specifically, Perrachione et al. (2016) reported peaks in the left posterior inferior frontal sulcus (pIFs; MNI coordinates: x = −39, y = 11, z = 26), left posterior dorsal superior temporal sulcus (pdSTS; MNI coordinates: x = −56, y = −39, z = 6), left pre-supplementary motor area (pre-SMA; MNI coordinates: x = −6, y = 10, z = 52), left occipital cortex (OC; MNI coordinates: x = −17, y = −76, z = 12), left posterior temporal fusiform gyrus (pTF; MNI coordinates: x = −38, y = −41, z = −19), left Putamen (MNI coordinates: x = −21, y = 7, z = 0), and right Putamen (MNI coordinates: x = 22, y = 12, z = −1) (see Fig. 4b). Note that the pTF corresponds to the anterior part of the visual word form area (VWFA)^39,40^.

**Fig. 5:**
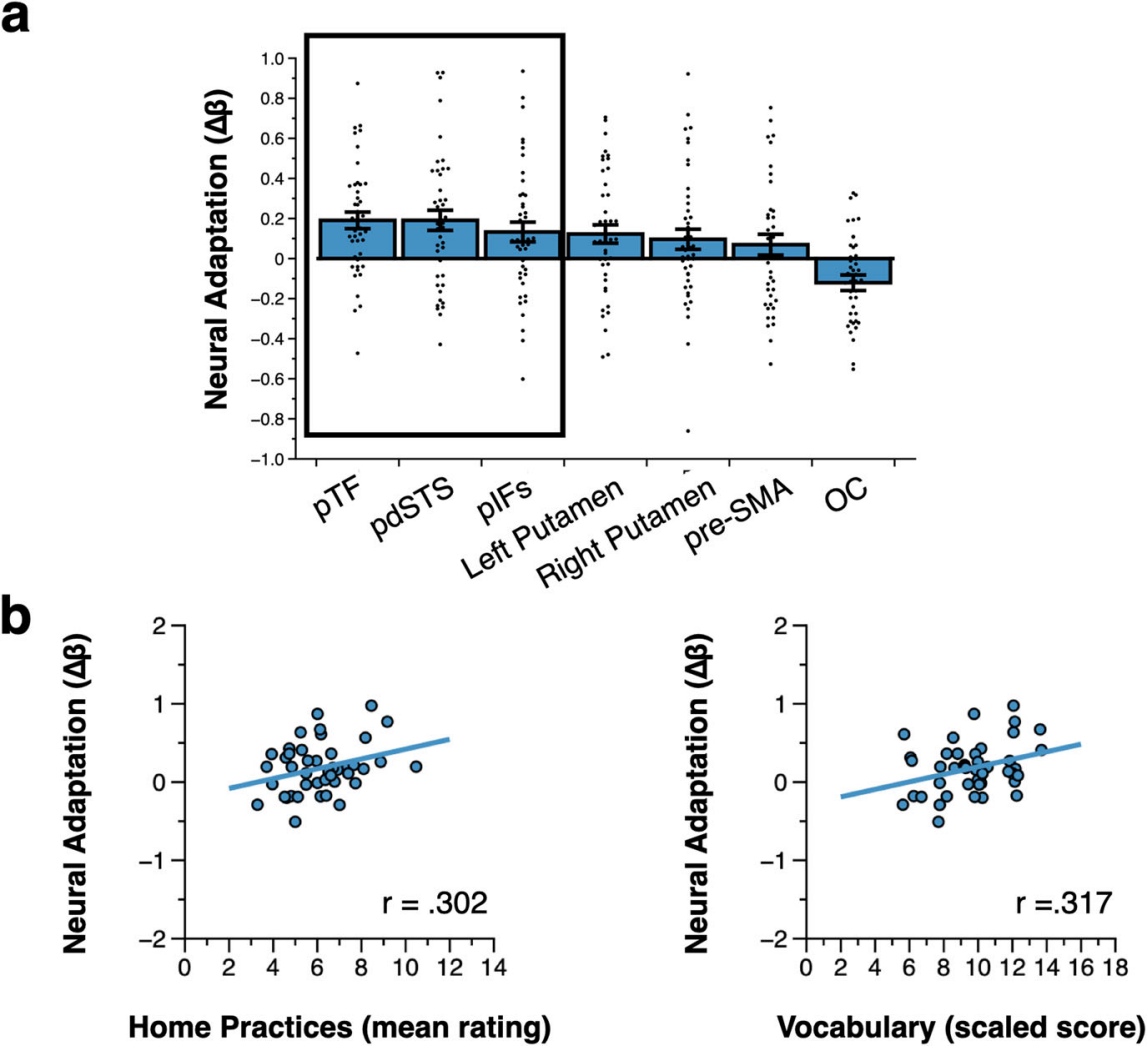
Neural adaptation to words (*n* = 44). **a** Average neural adaptation to words in the ROIs, ranked by effect size. The dark outline shows the brain regions for which word adaptation was significant after correction for multiple comparisons (excluding the left putamen, see text). Error bars represent the standard error of the mean (SEM). Each dot represents a participant. **b** Scatterplots of neural adaptation to words in the pIFs as a function of the overall frequency of home literacy practices (left) and child vocabulary (right). Analyses were controlled for parental income and education.

Averaging across all children from the fMRI sample (*n* = 44), word adaptation effects were significant after Bonferroni correction for multiple comparisons in three of these ROIs: the pIFs (*t*(43) = 2.698, *p*_corr_ = 0.035, *d* = 0.407), pdSTS (*t*(43) = 3.794, *p*_corr_ = 0.002, *d* = 0.572), and pTF (*t*(43) = 4.613, *p*_corr_ < 0.001, *d* = 0.695). The word adaptation effect, however, was only partially significant the putamen (left Putamen: *t*(43) = 2.674, *p*_corr_ = 0.037, *d* = 0.403, right Putamen: *t*(43) = 1.912, *p*_corr_ = 0.217, *d* = 0.288) and was not significant in the pre-SMA (*t*(43) = 1.335, *p*_corr_ = 0.658, *d* = 0.201) and the OC (word: *t*(43) = −3.103, *p*_corr_ > 0.999, *d* = −0.468) (see Fig. 5a).

We then tested our third prediction that the overall frequency of home literacy practices would be related to word adaptation effects in ROIs. We focused here on the ROIs for which we found significant word adaptation effects across all participants (excluding the putamen as a word adaptation effect was not uniformly found in that ROI). After correcting for multiple comparisons, there was a positive relation between frequency of home literacy practices and word adaptation in the pIFs (*r*(42) = 0.322, *p*_corr_ = 0.048), but not in the pdSTS (*r*(42) = 0.052, *p*_corr_ > 0.999) and pTF (*r*(42) = −0.056, *p*_corr_ > 0.999). The relation between frequency of home literacy practices and word adaptation remained significant in the pIFs after controlling for parental income and education (partial *r*(42) = 0.302, *p* = 0.026) (see Fig. 5b, left).

**Fig. 6:**
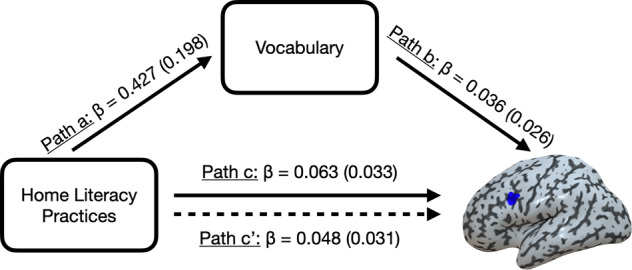
Mediation analysis (*n* = 44). The relation between home literacy practices and word adaptation in the pIFs was influenced by vocabulary (see main text). The solid line from home literacy practices to word adaptation in the pIFs represents the total effect (Path c). The dotted line represents the direct effect (Path c′). The analysis was controlled for parental income and education.

Finally, our fourth prediction was that the relation between HLE and either reading fluency or word adaptation would be influenced by children’s vocabulary skills. Because we did not find a relation between home literacy practices and reading fluency, we focused on word adaptation. First, we tested whether vocabulary was related to word adaptation effects in the pIFs, the only region in which there was a relation between activity and frequency of home literacy practices (see above). Controlling for parental income and education, there was a positive relation between vocabulary and word adaptation in the pIFs (partial *r*(42) = 0.317, *p* = 0.020) (see Fig. 5b, right). Therefore, the frequency of home literacy practices was related to both vocabulary and word adaptation in the pIFs. Vocabulary was then entered as a potential mediator of the relation between overall frequency of home literacy practices and word adaptation in the pIFs in a mediation analysis (see Methods and Fig. 6). As expected, the total relationship between home literacy practices and word adaptation was significantly positive, even in the fMRI sample (path c, coefficient = 0.063, standard error, or STE = 0.033, 95% CI Lower = 0.011, *p* = 0.024). Also as expected, more frequent home literacy practices were associated with improved vocabulary (path a, coefficient = 0.427, STE = 0.198, 95% CI Lower = 0.063, *p* = 0.024). Improved vocabulary also tended to be associated with larger word adaptation even after controlling for frequency of home literacy practices (path b, coefficient = 0.036, STE = 0.026, 95% CI Lower = −0.003, *p* = 0.062). Critically, the relation between frequency of home literacy practices and word adaptation was significantly mediated by vocabulary (path a*b, coefficient = 0.015, STE = 0.014, 95% CI Lower = 0.001, *p* = 0.046). Finally, the relation between frequency of home literacy practices and word adaptation was no longer significant after accounting for vocabulary (direct effect or path c′, coefficient = 0.048, STE = 0.031, 95% CI Lower = −0.001, *p* = 0.052).

### Additional analyses

**Fig. 7:**
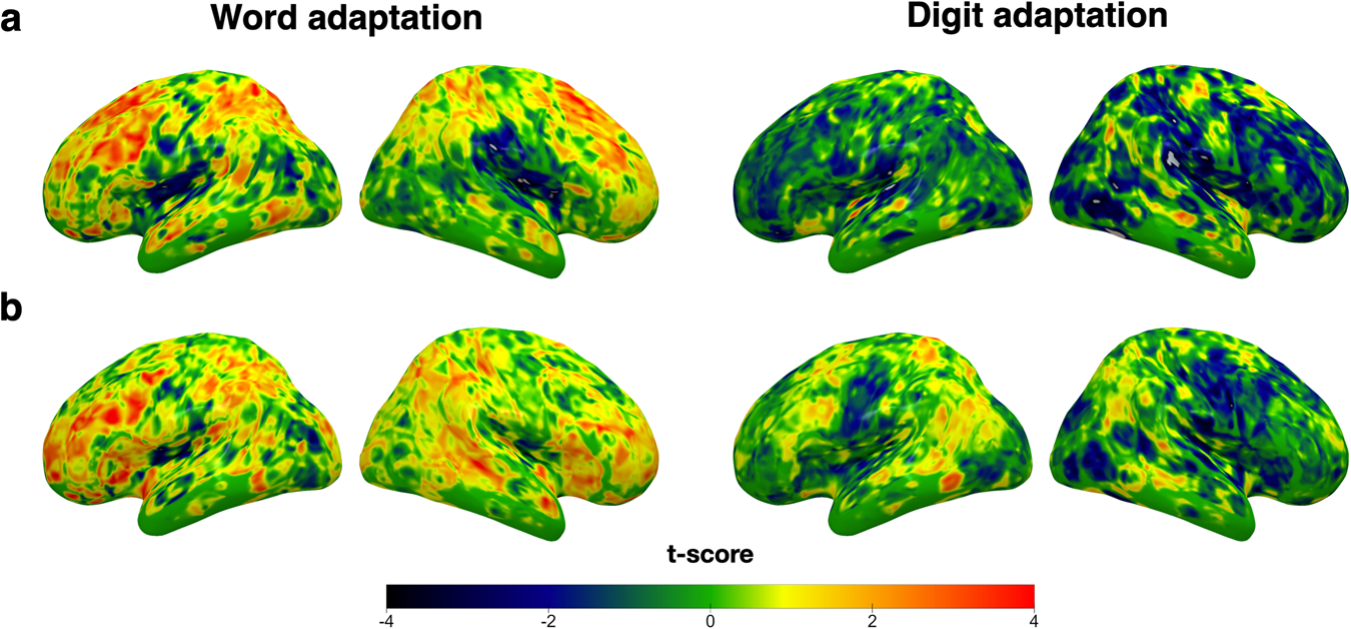
Unthresholded maps of *t* values showing the relations between neural adaptation, home literacy practices, and child vocabulary across the whole brain (*n* = 44). **a** Lateral views of the left and right hemispheres showing regions in which neural adaptation to words (left) and digits (right) increased with the overall frequency of home literacy practices. **b** Lateral views of the left and right hemispheres showing regions in which word adaptation (left) and digit adaptation (right) increased with child vocabulary.

We performed four sets of additional control analyses. First, to evaluate the specificity of our effects for print words, we measured the relation between adaptation to digits in the ROIs and both home literacy practices and vocabulary. There was no positive relation between digit adaptation and either frequency of home literacy practices or vocabulary in any of the seven ROIs (home literacy practices: all *r*’s < 0.004, all *p*’s > 0.493; vocabulary: all *r*’s < 0.188, all *p*’s > 0.117), including the pIFs (see Supplementary Fig. 1 and Supplementary Table 1). Figure 7 shows the unthresholded *t*-maps of the relation between either word or digit adaptation and home literacy practices and vocabulary across the whole brain.

Second, adaptation effects were measured using a passive task. Therefore, it is possible that individual differences in task engagement might influence our results. To test for this possibility, we embedded a target detection task among the stimuli (see Methods). On average, children detected 89% of targets (SD = 15) in the word adaptation task and 89% of targets (SD = 19) in the digit adaptation task. There was no difference in target detection rate between tasks (Wilcoxon rank-sum test: *p* = 0.730). Critically, target detection rates were not correlated with home literacy practices or vocabulary in any of the tasks (word adaptation: all rhos < −0.045, all *p*’s > 0.615; digit adaptation: all rhos < 0.114, all *p*’s > 0.231).

Third, although it has been hypothesized that home literacy practices are related to children’s reading through improved vocabulary, it is also possible that parents engage in more frequent literacy practices at home because they perceive that their child is skilled in reading (which may result in both improved vocabulary and enhanced brain sensitivity to print words). To tease apart these possibilities, we asked parents to indicate their perception of their child’s reading skill. This parental estimate was positively correlated with both reading accuracy (*r*(64) = 0.386, *p* < 0.001) and reading speed (*r*(64) = 0.534, *p* < 0.001), though it was not positively correlated with vocabulary (*r*(64) = 0.124, *p* = 0.161). However, this parental perception of reading skills was not correlated with home literacy practices (controlling for parental income and education, partial *r*(64) = 0.025, *p* = 0.421) or word adaptation in the pIFs (controlling for parental income and education, partial *r*(42) = 0.115, *p* = 0.229). When controlling for this parental estimate of child’s reading skill, literacy practices remained related to vocabulary (controlling for parental income and education, partial *r*(64) = 0.249, *p* = 0.022) and word adaptation in the pIFs (controlling for parental income and education, partial *r*(42) = 0.302, *p* = 0.023).

Finally, associations between literacy practices and children’s skills may also result (at least to some extent) from genetic predispositions to high academic achievement that parents may pass on to children. For instance, skilled parents might maintain relatively high-quality literacy environments^41^. Following Hart, Little, and Bergen^42^, we attempted to control for this effect by measuring parental reading fluency as a genetic proxy for predisposition to literacy skill. Critically, the relation between literacy practices and vocabulary remained significant after controlling for parent reading accuracy and reading speed (also controlling for parental income and education, partial *r*(64) = 0.248, *p* = 0.022).

## Discussion

Studies indicate large variations in the home literacy environment of children. Although these variations have been shown to relate to children’s reading skills, the relation between home literacy practices and brain activity underlying word-level reading remains unknown. The main goal of this study was to assess whether home literacy practices are associated with activity in brain regions supporting word recognition in 8-year-olds. We also aimed to test whether the relation between home literacy practices and activity associated with word recognition may be influenced by children’s vocabulary, as suggested by previous studies^22,29^.

First, based on prior literature, we predicted that the frequency of home literacy practices would be positively related to both children’s reading fluency and vocabulary skills. This hypothesis was only partially supported. We did find a relation between more frequent parental practices and enhanced children’s vocabulary, in keeping with a long line of previous studies on the HLE^4,6,43^. However, there was no significant relation between home literacy practices and any of our measures of reading fluency (accuracy or speed). Although this may be surprising given multiple reports of such a relation in the literature^5,8^, we could think of at least two reasons why we failed to find this association here. A first possibility is that the reading fluency test we used^36^ might have lacked sensitivity to discriminate between the reading fluency of our participants. Indeed, the Alouette test was initially designed to screen for dyslexia among children based on phonological-recoding skills^44^, which are early precursors of decoding skills. However, all of our participants were typical readers and it is possible that this 3-min test might not have been sensitive enough to capture variance within this population. A second possible reason is that the influence of the home environment on reading fluency skills may vary depending on children’s age^1,45^. Our participants were 8-year-olds who were already relatively efficient readers. For example, it is possible that individual differences in the reading fluency of these children might have been better captured by a reading comprehension task^46^. Note that we also did not find a relation between the frequency of home literacy practices and SES. Although this is inconsistent with several previous studies^15,16^, it is possible that this lack of relation might be due to the relatively high concentration of children from middle to high SES families in our sample.

Second, we assessed the replicability of our task by evaluating whether word adaptation effects would be found across participants in the same regions reported by Perrachione et al.^31^, who used the same task in a different population (adults) and language (English). Both whole-brain and ROI analyses showed that we largely replicated Perrachione et al.’s findings^31^. That is, word adaptation effects were found in a left-lateralized brain network that encompassed the inferior frontal, temporal, and occipital cortices. Not only are these regions located in the classic reading brain network^47,48^, these findings suggest that the task robustly assesses neural adaptation to print words in both French and English and generalizes across two different samples. This is significant given recent concerns about the reliability and replicability of fMRI studies^49^.

Third, we predicted that home literacy practices would be associated with activity in brain regions supporting word recognition. Somewhat surprisingly, we did not find any relation between home literacy practices and brain regions associated with visual processing, including the VWFA that has been shown to be specialized for letter strings^50^. Because this region develops during the first years of reading acquisition in children^51^, it is possible that VWFA activity during word recognition might be influenced by home literacy practices in younger children. In any case, our results suggest that home literacy practices may not strongly influence the visual processing of words in 8-year-olds. However, we found that home literacy practices were associated to word adaptation in the left pIFs in our sample of 8-year-olds. In other words, children benefiting from a higher quality home literacy environment had heightened sensitivity to the repetition of print words in an area located in the dorsal and posterior part of the left inferior frontal gyrus (IFG). This area, together with the larger adjacent left IFG pars triangularis, has consistently been associated with reading in both adults and children, as shown in a meta-analysis of studies involving reading or reading-related tasks with visual word, nonword, or letter string stimuli^50^. The specific role of this region - and of the larger anterior reading circuit—during reading remains unclear and may involve both phonological and semantic processing^52,53^. Nonetheless, there is evidence that reading skills are positively correlated with activity associated with print words in the pars triangularis in a sample of children from third and fourth grade^54^. Interestingly, several studies have previously found associations between the home literacy environment and the structure and function of the left IFG more broadly^21,25,28,30^. However, to our knowledge, none of these studies have directly measured activity associated with word-level reading in this region. Therefore, our study provides the first evidence that disparities in children’s home literacy environment may relate to functional differences in how the dorsal part of the left IFG processes words.

Fourth, based on prior literature^22,29^ we aimed to assess whether vocabulary may mediate the relation between home literacy practices and activity associated with word recognition. Not only did we find an association between children’s vocabulary skills and word adaptation in the left pIFs, our results indicate that the relation between home literacy practices and word adaptation in the left pIFS was mediated by children’s vocabulary. This is consistent with a model positing that children’s vocabulary skills mediate the relation between home literacy environment and mechanisms supporting word-level reading (see Fig. 1a). This is also consistent with previous behavioral studies showing that HLE effects on literacy outcomes may be mediated by earlier precursor skills, most notably vocabulary^3,29,55^. Overall, these findings support the general idea that the relation between the home environment and the brain mechanisms supporting reading in children is influenced by cognitive precursors to reading, such as vocabulary^22^ (see Fig. 1a).

Fifth, we anticipated that the relations between home literacy practices and neural adaptation would not be specific to words. Therefore, we also presented participants with an adaptation task in which words were replaced by Arabic numerals. We did not find any significant relation between home literacy practices and digit adaptation in any of our ROIs. There was also no significant relation between vocabulary and digit adaptation in the ROIs. Therefore, our effects cannot be easily explained by low-level perceptual adaptation effects or adaptation effects involving domain-general symbolic processing.

Finally, it is important to consider some limitations of this study. For example, our cross-sectional design does not allow us to formally test causal claims in Fig. 1a. Specifically, the relations that we observed are in line with this model. However, there may be other models that are compatible with our findings (e.g., the frequency of home literacy practices may increase in response to children’s reading skills). Our measure of the HLE also relies on a questionnaire. Not only may questionnaires be associated with a recall bias (i.e., parents may inaccurately or incompletely remember events that happened in the past), they might also be associated with a social desirability bias (i.e., parents may have reported more frequent literacy practices in an effort to avoid embarrassment and project a more favorable image to the experimenters)^56^. Finally, our study is restricted in terms of sample size, age range investigated, and reading task. Clearly, reading encompasses much more than word recognition and it is unclear to what extent our findings may apply to other age groups (e.g., home literacy practices may change with age) as well as to the relation between home literacy practices and brain mechanisms underlying reading comprehension or other higher-level reading processes.

We attempted to minimize these limitations in several ways. For instance, although our study is fundamentally correlational, the model we test here is based on extensive prior research on the HLE^5,11,22,29^. We also attempted to control for other plausible interpretations by gathering information about parental skills and perception of children’s reading skill (see Additional analyses). Even though recall and social desirability biases may still be present, we attempted to limit these biases by asking parents to report information about a very wide range of home activities and expectations (e.g., math, sport, music). Although our sample size is similar to that of previous studies that explored associations between home literacy practices and the reading network in the brain^26,28^, we adopted ROI analyses to maximize power^34^ (though a drawback of this approach is that it leaves open the possibility than other meaningful relations may be observed elsewhere in the brain). Notwithstanding these attempts to address limitations, it is clear that our results need to be substantiated by interventional studies in order to test the causal claims suggested here, as well as by future neuroimaging studies investigating the relation between home literacy practices and the reading brain in younger children and different tasks.

To conclude, we found that home literacy practices are associated with brain mechanisms supporting word recognition in 8-year-olds, in line with the idea that experiences beyond the classroom may influence brain regions supporting reading and learning^57^. Our results also support a model suggesting that home literacy practices may affect children’s reading through precursors skills such as vocabulary^10,11^. Nonetheless, our study was cross-sectional and the effects found were relatively small in size. Therefore, it calls for future longitudinal and interventional studies investigating how home literacy practices in younger children shape the development of the brain networks supporting reading.

## Methods

### Participants

Seventy-three right-handed children from second and third grade and one of their parents were recruited through flyers sent to schools and advertisements on social media. The experiment involved two sessions. In the first session, parents and children completed tests and questionnaires in the lab. In the second session, children completed the experimental tasks in the scanner. Seven children were excluded from analyses of the first session because they (1) were seeing a speech-language pathologist on a regular basis (*n* = 3), (2) had an intelligence quotient (IQ) lower than the 25th percentile (*n* = 2), (3) had a delay in speech and language acquisition (*n* = 1), and (4) were diagnosed with attention deficit disorder (*n* = 1). Therefore, 66 children were included in the behavioral sample. Out of the 58 children who participated the second (i.e., fMRI) session, 14 were excluded from the fMRI analyses because of incomplete data acquisition (*n* = 7) or excessive motion in the scanner (*n* = 7) (see criteria below). Therefore, our fMRI sample consisted of 44 children who had at least one run of data analyzable in both the word and digit adaptation tasks.

Children had a full-scale IQ comprised between 83 and 135 (mean = 112, standard deviation [SD] = 11), as measured by the NEMI-2 test^37^. A previous study^58^ explored the relation between brain activity and home numeracy practices in the same sample of participants. Demographic information about children and parents can be found in Table 2. All children and parents were native French speakers. Eighty-nine percent of the parents were mothers. Parental income ranged from less than €6000 to more than €60,000 per year (the median annual income in France is about €20,400;^59^). Fourteen percent of parents reported only having a secondary degree, 50% reported having an undergraduate degree, and 36% a master’s degree or higher. One parent did not go to high school. Therefore, SES ranged from relatively low to relatively high. Parents gave written informed consent and children gave their assent to participate in the study. The study was approved by a French ethics committee (Comité de Protection des Personnes Sud-Est 2). Families were paid 80 euros for their participation.

### Justification of fMRI sample size

The relation between children’s literacy skills and home literacy practices typically ranges between *r* = 0.40 to *r* = 0.60^30,60^. This range is in line with two previous studies that investigated the relation between home literacy practices and language-related (phonological processing and story listening) brain activity in young children. Specifically, Powers et al.^30^ and Romeo et al.^28^ (*n* = 36) found effect sizes of this relation ranging from *r* = 0.40 and *r* = 0.58 (see Table 5 in Powers et al.;^30^ and openly available dataset in Romeo et al.^28^). Considering these estimates, power analyses using G* Power version 3.1^61^ indicate that our final fMRI sample size of *n* = 44 would provide at least 88% power to detect correlations between home literacy practices and reading-related activity in the expected direction (α = 0.05, one-tailed). Because the present study also aims at investigating whether reading-related brain activity mediates the relation between home literacy practices and literacy skills, we performed a power analysis of such a mediation using the exact parameters reported by Romeo et al.^28^ in their mediation analysis involving language-related brain activity, conversional turns, and literacy skills. Monte Carlo simulations implemented in the online tool developed by Schoemann, Boulton, and Short^62^ (https://schoemanna.shinyapps.io/mc_power_med/) indicated a power of 86% to detect an influence of the mediator (i.e., brain activity) on the relation between the predictor (i.e., home literacy practices) and the outcome (i.e., literacy skills) based on Romeo et al.’s estimates (1000 iterations, 95% Confidence level). Overall, although it is important to note that this effect size is likely inflated because of publication bias^63^, our final fMRI sample size appears to be adequately powered to detect the hypothesized relations.

### Assessment of child and parent literacy skills

Children’s reading and vocabulary skills were assessed using the Alouette-R test^36^ and the “vocabulaire” subtest of the NEMI-2^37^. The Alouette-R test is a standardized test that is frequently used to test reading skills in French-speaking countries. This test requires participants to read a 265-word text aloud in 3 min. The number of words read and the number of pronunciation errors are used to calculate indices of reading speed and reading accuracy, respectively. The test has notably been shown to be a sensitive and specific screening tool detecting individuals with reading difficulties^44^. The NEMI-2 is a standardized IQ test that is similar to the Stanford–Binet Intelligence Scales. It uses measures of verbal intelligence (i.e., “general knowledge”, “vocabulary”, and “comparison” subtests) and matrix reasoning (i.e., “Raven’s matrices” subtest) to provide a composite IQ score that is highly correlated (*r* = 0.80) with the WISC score^37^. The “vocabulaire” subtest of the NEMI-2 requires children to define up to 27 words that decrease in frequency. The subtest is untimed and is stopped after three consecutive errors. Age-normalized reading and vocabulary scores are presented in Table 2. Parental reading skills were also tested using the Alouette-R test.

### Parental questionnaire

The frequency of home literacy practices was assessed using an electronic questionnaire given to parents on a tablet. After providing information about SES, parents were asked how often they engaged in home literacy activities with their children. Questions also concerned practices involving other academic and non-academic domains (e.g., math, music, biology). These questions, which helped reduce the focus on literacy practices, are considered fillers in the present study (items about math practices are analyzed in Girard et al.^58^). Practices were adapted from the questionnaire used by Skwarchuk and colleagues^38^. Using a six-point rating scale (Did not occur/Activity is not relevant to my child, 1–3 times per month, Once per week, 2–4 times per week, almost daily, daily), parents rated the frequency with which they engaged in that practice with their child at home during the past month. Parents also had the option to indicate whether the practice occurred in the past but no longer at the time of testing (rated 1 point) and whether the child engaged in the practice at home but on her own (also rated 1 point).

Among the 77 questions, 18 concerned home literacy practices. These included a mixture of informal and formal practices (Fig. 2). As defined in Sénéchal and LeFevre (p. 1552^5^), “informal literacy experiences are those where the print is present but is not the focus of the parent–child interaction. In contrast, formal literacy activities are those where the attention is on the print itself”. Formal practices were of varying difficulty, encompassing both basic or more advanced activities for an 8-year-old child. For example, listening when children read out loud was considered relatively basic but asking questions when children read was considered more advanced. Because some advanced activities were inherently related to more basic activities (e.g., reading/writing short texts and reading/writing long texts), those advanced items were presented to parents only if they stated that the corresponding more basic activity had occurred at home. This was done to avoid parents having to repeatedly say no to an activity they had never engaged in). Ratings associated with frequencies of home literacy practices are shown in Fig. 2. Frequencies of activities were averaged across items within each category to gather separate scores of informal, formal basic, and formal advanced practices. We also used a composite score, corresponding to the sum of these three subscores. Finally, parents were asked to provide a subjective estimate of their child’s skills in reading (and other domains) using a six-point rating scale (i.e., Not sure, Severe difficulty, Difficulty, Average skills, Good skills, Very good skills).

### Experimental tasks

We adapted from Experiment 2B of Perrachione et al.^31^ a task in which a series of words were passively presented in blocks at the center of the screen (see Fig. 1b). As in Perrachione et al.^31^, words were monosyllabic nouns of three to five letters in length (mode = 4). The only difference between our task and that of Perrachione et al.^31^ was that our stimuli were in French instead of English. Words were thematically varied and equated for frequency using the CHACQFAM database in http://www.lexique.org/shiny/openlexicon/. In another version of the task that served as a control in the present study, words were replaced by Arabic digits ranging from 1 to 8. Word and digit adaptation tasks were each composed of two runs. In each of these runs, participants were presented with adaptation and no-adaptation blocks. Adaptation blocks consisted of the repetition of the same stimulus (word or digit) eight times. No-adaptation blocks consisted of the presentation of eight different stimuli. Another task involving dot arrays was included in the scanning session but is not analyzed here.

### fMRI data acquisition

Images were collected using a Siemens Prisma 3 T MRI scanner with a transmit body coil and a 64-channel receiver head-neck coil (Siemens Healthcare, Erlangen, Germany) at the CERMEP Imagerie du vivant in Lyon, France. The BOLD signal was measured with a susceptibility-weighted single-shot EPI sequence. Imaging parameters were as follows: TR = 2000 ms, TE = 24 ms, flip angle = 80°, matrix size = 128 × 120, field of view = 220 × 206 mm, slice thickness = 3 mm (0.48 mm gap), number of slices = 32. A high-resolution T1-weighted whole-brain anatomical volume was also collected for each participant. Parameters were as follows: TR = 3500 ms, TE = 2.24 ms, flip angle = 8°, matrix size = 256 × 256, field of view = 224 × 224 mm, slice thickness = 0.9 mm, number of slices = 192.

### Experimental timeline

The experimental timeline, which was directly taken from Perrachione et al.^31^, was identical in both the word and digit adaptation task. In each block, stimuli remained on the screen for 700 ms, with a 500 ms inter-stimulus interval (for a total block duration of 9.6 s). Ten adaptation blocks and ten no-adaptation blocks were presented along with ten blocks of visual fixation (duration = 9.6 s) in each run. Block presentation was pseudo-randomized such that two blocks of the same type could not follow each other. Finally, ten target stimuli (a picture of a rocket) randomly appeared in each run (outside of blocks). Participants were asked to press a button every time this target appeared.

### fMRI data preprocessing

Images were analyzed with SPM12 (Wellcome Department of Cognitive Neurology, London, UK). The first four images of each run were discarded to allow for T1 equilibration effects. Functional images were corrected for slice acquisition delays and spatially realigned to the first image of the first run to correct for head movements. Realigned images were smoothed with a Gaussian filter (4 mm × 4 mm × 7 mm full-width at half maximum). Using ArtRepair (https://cibsr.stanford.edu/tools/human-brain-project/artrepair-software.html), functional volumes with a global mean intensity greater than three standard deviations from the average of the run or a volume-to-volume motion greater than 2 mm were identified as outliers and substituted by the interpolation of the two nearest non-repaired volumes^28^. Participants with outliers in more than 20% of volumes were excluded from the analyses (*n* = 7). After outlier exclusion, the movement range was on average 0.01 (SD = 0.04), 0.17 (SD = 0.30), and 0.18 (SD = 0.64) mm in the x, y, and z direction, with 0.27 (SD = 0.87), 0.37 (SD = 1.17), 0.45 (SD = 1.46) degrees of roll, pitch, and yaw.

Finally, functional images were normalized into the same stereotaxic space. Studies have found that anatomical differences between children older than 7–8-year-olds and adults are small enough that they are beyond the resolution of fMRI experiments^64,65^. Therefore, considering the age of our participants, the resolution of our data, and the fact that we wanted to be able to compare our results to that from Perrachione et al.^31^ with adult participants, we normalized all individual brains into the standard adult Montreal Neurological Institute (MNI) space. This was done in two steps. First, after coregistration with the functional data, the structural image was segmented into gray matter, white matter, and cerebrospinal fluid by using a unified segmentation algorithm^66^. Second, the functional data were normalized to the MNI space by using the normalization parameters estimated during unified segmentation (normalized voxel size, 2 mm^3^ × 2 mm^3^ × 3.5 mm^3^).

### fMRI data analysis

Statistical analysis of fMRI data was performed according to the GLM. Brain activity associated with periods of adaptation and no-adaptation was modeled as epochs with onsets time-locked to the beginning of each block and a duration of 9.6 s. All epochs were convolved with a canonical hemodynamic response function. The time-series data were high-pass filtered (1/128 Hz), and serial correlations were corrected using an auto-regressive AR(1) model.

For each participant, the word adaptation effect was identified by subtracting activity associated with adaptation blocks from activity associated with no-adaptation blocks. Individual contrasts were then submitted to one-sample *t*-tests across all participants. First, we used a whole-brain voxelwise approach to evaluate whether the overall brain network involved in word adaptation was similar to that found in Perrachione et al.^31^. An FDR-corrected cluster-level threshold of *p* < 0.05 (defined using voxel-level thresholds of *p* < 0.001 and *p* < 0.002) was applied to the whole-brain statistical map to assess brain activations. Second, we used an a priori region of interest (ROI) approach to measure activity in the seven brain regions that showed a word adaptation effect (i.e., lower activity in adaptation than no-adaptation blocks of the word adaptation task) in normal adults in Perrachione et al.^31^ (see Fig. 3). All ROIs were 6-mm radius spheres centered on coordinates reported in Table S5 for Experiment 2B of Perrachione et al.^31^. We tested for the presence of a word adaptation effect in each ROI using a series of one-sample *t*-tests. *P* values were Bonferroni-corrected for multiple comparison. In ROIs for which there was a significant word adaptation effect, the neural adaptation effect was then correlated with the frequency of home literacy practices. Mediation analyses were then performed to test whether, in regions for which there was a relation between home literacy practices and word adaptation, there was a mediating effect of vocabulary. These analyses were performed using the M3 toolbox in Matlab (https://github.com/canlab/MediationToolbox^67^). Briefly, a mediation estimates the degree to which a variable (e.g., M) can explain the relation between two other variables (e.g., X and Y). Here, X was defined as the frequency of home literacy practices. Y was defined as the word adaptation effect. M was defined as vocabulary. Path a reflected the relation between home literacy practices and vocabulary. Path b reflected the relation between vocabulary and word adaptation, controlling for home literacy practices. The total relationship between frequency of home literacy practices and word adaptation (including direct and indirect effects) is reflected in path c, while the direct effect of the relationship between frequency of home literacy practices and word adaptation (controlling for vocabulary) is reflected in path c′. Finally, the significance of the mediator was tested in the product a*b^67–69^. A bias-corrected bootstrap test over 10,000 iterations was used to measure the statistical significance of the mediator. Mediation analyses systematically included as covariates parental education and income to control for differences in SES between children.

Because our hypotheses were directional (i.e., we only anticipated lesser activity in adaptation than no-adaptation blocks, as well as increases in either literacy skills or neural adaptation effects as a function of home literacy practices), *p* values for behavioral and fMRI results are one-sided (unless otherwise noted). Accordingly, only 95% lower bound confidence intervals (95% CI Lower) are reported.

### Reporting Summary

Further information on research design is available in the Nature Research Reporting Summary linked to this article.

## Supplementary information

Supplementary Information

Reporting Summary

## Data Availability

The task, electronic version of the questionnaire (in French), behavioral data, and individual MRI data are available from Zenodo (10.5281/zenodo.5112814). The ROI images and the whole-brain unthresholded p-maps corresponding to Fig. 4a and Fig. 7 are available from NeuroVault (https://identifiers.org/neurovault.collection:9453).

## Code availability

The Matlab scripts used to analyze fMRI data are available from GitHub (https://github.com/BBL-lab/BBL-batch-system). The M3 toolbox is also available from GitHub (https://github.com/canlab/MediationToolbox).
