# Supplementary material for: Nurturing the reading brain: home literacy practices are associated with children’s neural response to printed words through vocabulary skills
Source: NPJ Sci Learn. 2021 Dec 3;6:34. doi: 10.1038/s41539-021-00112-9 (PMC8642429; doi:10.1038/s41539-021-00112-9)
Supplement: Supplementary file 1 — Supplementary Information [file 41539_2021_112_MOESM1_ESM.pdf]

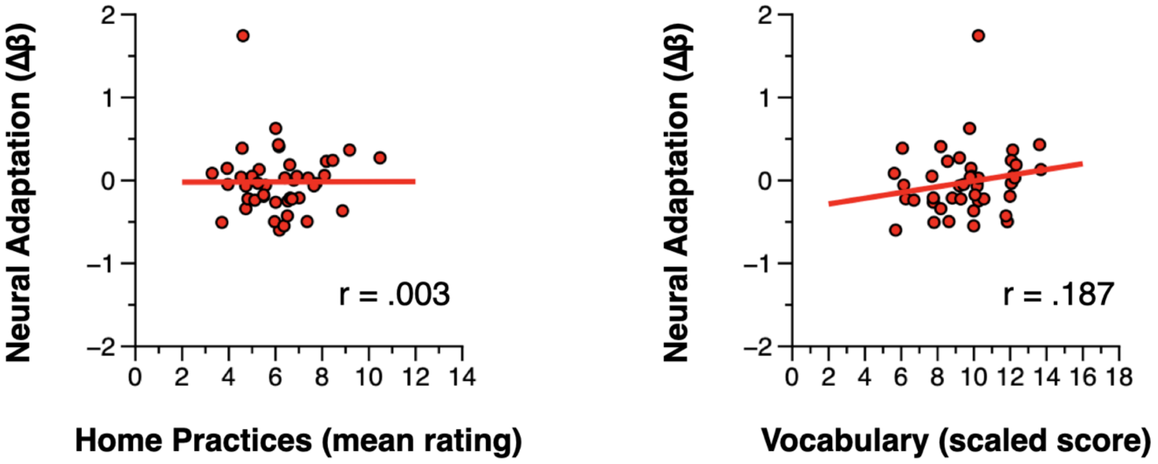

Supplementary Figure 1. Scatterplots of neural adaptation to digits in the pIFs as a function of the overall frequency of home literacy practices (left) and child vocabulary (right). Analyses were controlled for parental income and education.

Supplementary Table 1. Pearson correlation coefficients between home literacy practices, child vocabulary, and digit adaptation in all ROIs

	**1**	**2**	**3**	**4**	**5**	**6**	**7**	**8**	**9**
1- Literacy practices	—								
2- Vocabulary	**0.295**	—							
3- Left pIFs	0.003	0.187	—						
4- Left pdSTS	-0.177	0.068	**0.480**	—					
5- Left pre-SMA	-0.146	-0.012	**0.699**	**0.528**	—				
6- Left OC	-0.182	-0.012	0.049	0.242	0.212	—			
7 -Left pTF	-0.296	-0.009	**0.452**	**0.470**	**0.525**	**0.350**	—		
8- Left Putamen	-0.049	-0.123	**0.592**	**0.562**	**0.647**	0.108	**0.511**	—	
9- Right Putamen	-0.077	-0.186	**0.547**	**0.476**	**0.558**	0.161	**0.414**	**0.892**	—

Notes. Coefficients are adjusted for parental income and education. Values in bold are significant ($p < .05$, one-tailed)
