# Supplementary material for: Nurturing the reading brain: home literacy practices are associated with children’s neural response to printed words through vocabulary skills
Source: NPJ Sci Learn. 2021 Dec 3;6:34. doi: 10.1038/s41539-021-00112-9 (PMC8642429; doi:10.1038/s41539-021-00112-9)
Supplement: Supplementary file 2 — Reporting Summary [file 41539_2021_112_MOESM2_ESM.pdf]

nature portfolio

Corresponding author(s): Cléa Girard and Jérôme Prado

Last updated by author(s): Jul 20, 2021

Reporting Summary

Nature Portfolio wishes to improve the reproducibility of the work that we publish. This form provides structure for consistency and transparency in reporting. For further information on Nature Portfolio policies, see our Editorial Policies and the Editorial Policy Checklist.

Statistics

For all statistical analyses, confirm that the following items are present in the figure legend, table legend, main text, or Methods section.

n/a	Confirmed	
☐	☒	The exact sample size (n) for each experimental group/condition, given as a discrete number and unit of measurement
☐	☒	A statement on whether measurements were taken from distinct samples or whether the same sample was measured repeatedly
☐	☒	The statistical test(s) used AND whether they are one- or two-sided<br>*Only common tests should be described solely by name; describe more complex techniques in the Methods section.*
☐	☒	A description of all covariates tested
☐	☒	A description of any assumptions or corrections, such as tests of normality and adjustment for multiple comparisons
☐	☒	A full description of the statistical parameters including central tendency (e.g. means) or other basic estimates (e.g. regression coefficient) AND variation (e.g. standard deviation) or associated estimates of uncertainty (e.g. confidence intervals)
☐	☒	For null hypothesis testing, the test statistic (e.g. F, t, r) with confidence intervals, effect sizes, degrees of freedom and P value noted<br>*Give P values as exact values whenever suitable.*
☒	☐	For Bayesian analysis, information on the choice of priors and Markov chain Monte Carlo settings
☒	☐	For hierarchical and complex designs, identification of the appropriate level for tests and full reporting of outcomes
☐	☒	Estimates of effect sizes (e.g. Cohen's d, Pearson's r), indicating how they were calculated

Our web collection on statistics for biologists contains articles on many of the points above.

Software and code

Policy information about availability of computer code

Data collection	fMRI data were collected with a Siemens Prisma 3T MRI scanner (Siemens Healthcare, Erlangen, Germany) at the CERMEP Imagerie du vivant in Lyon, France.
Data analysis	fMRI data were analyzed with SPM12 https://www.fil.ion.ucl.ac.uk/spm/software/spm12/) with the ArtRepair toolbox https://cibsr.stanford.edu/tools/human-brain-project/artrepair-software.html). Custom Matlab scripts were used (https://github.com/BBL-lab/BBL-batch-system). We also used the M3 toolbox for the mediation analysis (https://github.com/canlab/MediationToolbox).

For manuscripts utilizing custom algorithms or software that are central to the research but not yet described in published literature, software must be made available to editors and reviewers. We strongly encourage code deposition in a community repository (e.g. GitHub). See the Nature Portfolio guidelines for submitting code & software for further information.

Data

Policy information about availability of data

All manuscripts must include a data availability statement. This statement should provide the following information, where applicable:

- Accession codes, unique identifiers, or web links for publicly available datasets
- A description of any restrictions on data availability
- For clinical datasets or third party data, please ensure that the statement adheres to our policy

The task, electronic version of the questionnaire (in French) and all behavioral data are publicly available via Zenodo at [http://doi.org/10.5281/zenodo.5112814]. The ROI images and the whole-brain unthresholded p-maps corresponding to Fig. 4A and Fig. 7 are available in NeuroVault, [https://neurovault.org/collections/XWKJNYOX/]. Finally, individual MRI data are available via Zenodo at [http://doi.org/10.5281/zenodo.5112814].

Field-specific reporting

Please select the one below that is the best fit for your research. If you are not sure, read the appropriate sections before making your selection.

☐ Life sciences ☒ Behavioural & social sciences ☐ Ecological, evolutionary & environmental sciences

For a reference copy of the document with all sections, see nature.com/documents/nr-reporting-summary-flat.pdf

Behavioural & social sciences study design

All studies must disclose on these points even when the disclosure is negative.

Study description	This study includes questionnaires, behavioral tests and fMRI data. Therefore, the study involves quantitative data.
Research sample	Seventy-three right-handed children from 2nd and 3rd grade and one of their parents participated in the study. The experiment involved two sessions. In the first session, parents and children completed tests and questionnaires in the lab. In the second session, children completed the experimental tasks in the scanner.
Sampling strategy	Children and parents were recruited through flyers sent to schools and advertisements on social media.
Data collection	Questionnaires were administered to parents using a tablet. Behavioral tests were administered in a testing room in the lab. fMRI data were collected with a Siemens Prisma 3T MRI scanner (Siemens Healthcare, Erlangen, Germany) at the CERMEP Imagerie du vivant in Lyon, France.
Timing	The data collection for this study started in February 2018 and continued up until December 2018.
Data exclusions	Seven children were excluded from analyses of the first session because they (1) were seeing a speech-language pathologist on a regular basis (n=3), (2) had an intelligence quotient (IQ) lower than the 25th percentile (n=2), (3) had a delay in speech and language acquisition (n=1), and (4) were diagnosed with attention deficit disorder (n=1). Therefore, 66 children were included in the behavioral sample. Out of the 58 children who participated the second (i.e., fMRI) session, 14 were excluded from the fMRI analyses because of incomplete data acquisition (n=7) or excessive motion in the scanner (n=7). Therefore, our fMRI sample consisted of 44 children who had at least one run of data analyzable in both the word and digit adaptation tasks.
Non-participation	No participants dropped out from the study.
Randomization	No randomization was necessary for this study

Reporting for specific materials, systems and methods

We require information from authors about some types of materials, experimental systems and methods used in many studies. Here, indicate whether each material, system or method listed is relevant to your study. If you are not sure if a list item applies to your research, read the appropriate section before selecting a response.

Materials & experimental systems

n/a	Involved in the study	
☒	☐	Antibodies
☒	☐	Eukaryotic cell lines
☒	☐	Palaeontology and archaeology
☒	☐	Animals and other organisms
☐	☒	Human research participants
☒	☐	Clinical data
☒	☐	Dual use research of concern

Methods

n/a	Involved in the study	
☒	☐	ChIP-seq
☒	☐	Flow cytometry
☐	☒	MRI-based neuroimaging

Human research participants

Policy information about studies involving human research participants

Population characteristics	The behavioral sample consisted in 66 French-speaking children from age 7.52 to 9.22 (mean = 8.46), while the fMRI sample consisted in 44 of these participants (age range = 8.02 – 9.14; mean = 8.49). SES ranged from relatively low to relatively high in both samples. Age-normalized scores for reading fluency and vocabulary skills (assessed using the Alouette-R test36 and the “vocabulaire” subtest of the NEMI-2 test37) were in the normal to superior range in both samples. IQ was also in the normal to superior range. Parental reading scores were in the normal range.
Recruitment	Seventy-three right-handed children from 2nd and 3rd grade and one of their parents were recruited through flyers sent to schools and advertisements on social media.

Ethics oversight | The study was approved by a French ethics committee (Comité de Protection des Personnes Sud-Est 2).

Note that full information on the approval of the study protocol must also be provided in the manuscript.

Magnetic resonance imaging

Experimental design

Design type: Block design

Design specifications: Experimental timeline was identical in both the word and digit adaptation task. In each block, stimuli remained on the screen for 700 ms, with a 500 ms inter-stimulus interval (for a total block duration of 9.6 seconds). Ten adaptation blocks and 10 no-adaptation blocks were presented along with 10 blocks of visual fixation (duration = 9.6 s) in each run. Block presentation was pseudo-randomized such that 2 blocks of the same type could not follow each other.

Behavioral performance measures: 10 target stimuli (a picture of rocket) randomly appeared in each run (outside of blocks). Participants were asked to press a button every time this target appeared. The detection score was used to know how participants payed attention to the stimuli in both tasks.

Acquisition

Imaging type(s): EPI and T1

Field strength: 3 Tesla

Sequence & imaging parameters: The BOLD signal was measured with a susceptibility weighted single-shot EPI sequence. Imaging parameters were as follows: TR = 2000 ms, TE = 24 ms, flip angle = 80°, matrix size = 128 × 120, field of view = 220 × 206 mm, slice thickness = 3 mm (0.48 mm gap), number of slices = 32. A high-resolution T1-weighted whole-brain anatomical volume was also collected for each participant. Parameters were as follows: TR = 3500 ms, TE = 2.24 ms, flip angle = 8°, matrix size = 256 × 256, field of view = 224 × 224 mm, slice thickness = 0.9 mm, number of slices = 192.

Area of acquisition: whole-brain

Diffusion MRI ☐ Used ☒ Not used

Preprocessing

Preprocessing software: Images were analyzed with SPM12 (Wellcome department of Cognitive Neurology, London, UK). The first 4 images of each run were discarded to allow for T1 equilibration effects. Functional images were corrected for slice acquisition delays and spatially realigned to the first image of the first run to correct for head movements. Realigned images were smoothed with a Gaussian filter (4 × 4 × 7 mm full-width at half maximum).

Normalization: Functional images were normalized into the standard adult Montreal Neurological Institute (MNI) space. First, after coregistration with the functional data, the structural image was segmented into grey matter, white matter, and cerebrospinal fluid by using a unified segmentation algorithm. Second, the functional data were normalized to the MNI space by using the normalization parameters estimated during unified segmentation (normalized voxel size, 2 × 2 × 3.5 mm3).

Normalization template: MNI

Noise and artifact removal: We used ArtRepair (https://cibsr.stanford.edu/tools/human-brain-project/artrepair-software.html) to identify volumes with motion artifacts.

Volume censoring: Functional volumes with a global mean intensity greater than 3 standard deviations from the average of the run or a volume-to-volume motion greater than 2 mm were identified as outliers and substituted by the interpolation of the 2 nearest non-repaired volumes. Participants with outliers in more than 20% of volumes were excluded from the analyses

Statistical modeling & inference

Model type and settings: Statistical analysis of fMRI data was performed according to the GLM. Brain activity associated with periods of adaptation and no-adaptation was modeled as epochs with onsets time-locked to the beginning of each block and a duration of 9.6 s. All epochs were convolved with a canonical hemodynamic response function. The time series data were high-pass filtered (1/128Hz), and serial correlations were corrected using an auto-regressive AR(1) model.

Effect(s) tested: For each participant, the word adaptation effect was identified by subtracting activity associated with adaptation blocks from activity associated with no-adaptation blocks. Individual contrasts were then submitted to one-sample t-tests across all participants. First, we used a whole-brain voxelwise approach. A FDR-corrected cluster-level threshold of $p < 0.05$ (defined using voxel-level thresholds of $p < .001$ and $p < .002$) was applied to the whole-brain statistical map to assess brain activations. Second, we used an a priori Region of Interest (ROI) approach to measure activity in the 7 brain regions that showed a word adaptation effect (i.e., lower activity in adaptation than no-adaptation blocks of the word adaptation task) in normal adults in Perrachione et al. All ROIs were 6-mm radius spheres centered on coordinates reported in Table S5 for Experiment 2B of Perrachione et al. We tested for the presence of a word adaptation effect in each ROI using a series of one-

sample t-tests. P values were Bonferroni-corrected for multiple comparison. In ROIs for which there was a significant word adaptation effect, the neural adaptation effect was then correlated with the frequency of home literacy practices. Mediation analyses were then performed to test whether, in regions for which there was a relation between home literacy practices and word adaptation, there was a mediating effect of vocabulary. These analyses were performed using the M3 toolbox in Matlab (https://github.com/canlab/MediationToolbox).

Specify type of analysis: ☐ Whole brain ☐ ROI-based ☒ Both

Anatomical location(s): We used an a priori Region of Interest (ROI) approach to measure activity in the 7 brain regions that showed a word adaptation effect (i.e., lower activity in adaptation than no-adaptation blocks of the word adaptation task) in normal adults in Perrachione et al. (2016, see Fig. 3B). All ROIs were 6-mm radius spheres centered on coordinates reported in Table S5 for Experiment 2B of Perrachione et al (2016)

Statistic type for inference (See Eklund et al. 2016): cluster-level correction

Correction: A FDR-corrected cluster-level threshold of $p < 0.05$ (defined using voxel-level thresholds of $p < .001$ and $p < .002$) was applied to the whole-brain statistical map to assess brain activations. For ROIs, we tested for the presence of a word adaptation effect in each ROI using a series of one-sample t-tests. P values were Bonferroni-corrected for multiple comparison

Models & analysis

n/a	Involved in the study
☒	☐ Functional and/or effective connectivity
☒	☐ Graph analysis
☒	☐ Multivariate modeling or predictive analysis
